# A Scaffold‐Based 3D Culture Model Including Selected Osteoprogenitors for Bone Regeneration With Controlled Morphology

**DOI:** 10.1155/sci/1860064

**Published:** 2026-04-10

**Authors:** Thi Hue Mai, Shousaku Itoh, Takumi Kagioka, Makato Abe, Mikako Hayashi

**Affiliations:** ^1^ Department of Restorative Dentistry and Endodontology, Graduate School of Dentistry, The University of Osaka, Osaka, Japan, osaka-u.ac.jp; ^2^ Department of Oral Anatomy and Developmental Biology, Graduate School of Dentistry, The University of Osaka, Osaka, Japan, osaka-u.ac.jp

**Keywords:** 3D culture, bone morphology, bone regeneration, osteoblasts, paracrine signaling, skeletal stem cells

## Abstract

We previously identified “highly purified osteoprogenitors” (HipOPs) as a promising model for studying bone organ regeneration in murine systems. However, achieving proper integration of the bone formed by HipOPs into the well‐organized host bone requires better morphological control. In this study, we developed a 3D culture model by culturing a HipOP‐seeded gelatin sponge on an osteoblast (OB) layer to regulate the morphology of regenerated bone. In vitro studies revealed significant upregulation of genes associated with bone formation, response to stimuli, cell communication, and vascularization in the HipOP population after 48 h of coculture. Moreover, the HipOPs exhibited concentration‐dependent responses, with stronger effects observed in regions closer to the OB layer. By day 7, the sponge areas in contact with the OB layer contained more cells with enhanced osteogenic differentiation capacity. When transplanted subcutaneously into immunodeficient mice for 8 weeks, the 3D culture samples formed bone organs comprising bone and bone marrow tissue. Notably, cortical bone appeared only in regions where the sponge had contacted the OB layer, with a gradual decrease in bone density observed outward from that area. Our 3D culture model successfully generated hierarchically structured bone organs, offering significant potential for reconstructing well‐integrated bone in defective regions.

## 1. Introduction

The bone is hierarchically structured, exhibiting a radial gradient of porosity that transitions from the dense cortical bone on the outside to the spongy bone on the inside. Mimicking this structural complexity remains a key challenge in bone engineering. Previous attempts to guide bone regeneration used scaffolds with graded porosity or particle size [1–4]. However, these approaches faced significant limitations due to intricate fabrication processes and inconsistent results, as the scaffolds provided only physical cues for the cells [3, 5].

Bone marrow mesenchymal stem cells (BMSCs) are multipotent, capable of differentiating into various cell types, including bone‐forming cells or osteoblasts (OBs). The fate of BMSCs is influenced by interactions with tissue‐specific cells such as chondrocytes and myocytes [6, 7]. In the endosteal niche near the internal bone surface, the synergy between bone‐lining OBs and their progenitors (BMSCs) remains poorly understood due to the native microenvironment’s complexity [8]. However, studies indicated that OBs influence BMSCs’ osteogenic differentiation through both direct contact [9, 10] and paracrine signaling [11, 12].

Signaling molecules secreted by OBs offer a promising chemical mechanism for controlling BMSC differentiation and bone formation. While coculturing OBs and BMSCs in 2D or 3D models has been shown to enhance osteogenesis [10, 13, 14], the effects of an OB layer on 3D‐cultured BMSCs remain unclear. We hypothesize that soluble molecules produced by OBs diffuse along a concentration gradient, inducing graded osteogenic differentiation in BMSCs within a 3D culture system and resulting in varying bone density.

Human BMSCs exhibit significant donor variation [15–17], posing challenges for consistent experimental results. To overcome this, BMSCs can be harvested from closed populations of inbred animal models, such as mice, which are widely used in biomedical research due to their small size, genetic similarity to humans, and ease of handling. However, the original method of collecting BMSCs from bone marrow tissue based on their plastic adherence was inefficient in mice [18, 19] compared to humans [20] and other species [21–23]. Untreated bone marrow cells from mice often contain high proportions of hematopoietic cells that may interfere with BMSC functionality [24]. To reduce contamination by hematopoietic cells, we previously cultured BMSCs for 14 days and then excluded the lineage‐committed hematopoietic cells using magnetic microbeads [19]. This simple strategy resulted in significant enrichment of skeletal stem cells with the capacity for OB, chondrocyte, and adipocyte differentiation in vitro. Although comprising multiple precursor cell types, the population is enriched more than 100‐fold in osteoprogenitors by limiting dilution assay, leading us to refer to the cells as “highly purified osteoprogenitors” (HipOPs) [19]. In addition to retaining the multipotent characteristics of BMSCs, HipOPs demonstrate superior stem cell markers by flow cytometry and the ability to reconstitute bone tissue and the bone marrow microenvironment by transplantation experiments [19]. However, the morphology of bone tissue formed by HipOPs was unnatural and would not allow good integration with well‐organized host bone.

This study introduces a new 3D culture model leveraging the effects of the OB layer to regulate the osteogenic differentiation of HipOPs, enabling controlled bone regeneration with tailored morphological outcomes.

## 2. Materials and Methods

### 2.1. Ethics Statement

This study was approved by the Research Ethics Committee of Graduate School of Dentistry, The University of Osaka. All experiments were practiced according to the committee’s guidelines related to animal care (R‐01‐001‐0) and were compliant with the guidelines of ARRIVE guidelines (https://arriveguidelines.org).

### 2.2. Overview of In Vitro and In Vivo Experiments

**Figure 1:**
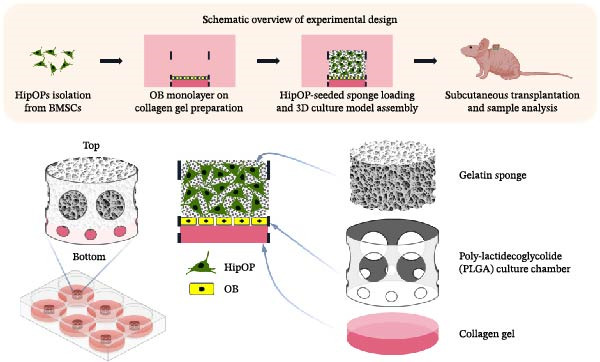
Design of the 3D culture model. The scaffolds included a collagen layer (2 mm thick) supporting a monolayer of osteoblasts (OBs), a cylindrical gelatin sponge (5 mm in diameter and 3 mm thick) for seeding highly purified osteoprogenitors (HipOPs), and a tubular poly‐lactidecoglycolide (PLGA) culture chamber (5 mm in diameter and 5 mm thick) with perforated holes for structural support. The cell interactions involve the OB monolayer and 3D‐cultured HipOPs. The models were cultured for 48 h before subcutaneous transplantation into immunodeficient mice.

Mouse BMCSs were purified through negative cell sorting to isolate HipOPs for subsequent experiments. Initially, HipOPs were utilized to generate a confluent monolayer of OBs on a collagen gel, with differentiation monitored to ensure the OB layer reached the appropriate stage for starting OB‐HipOP coculture. The responses of HipOPs in the coculture system were analyzed to determine the impact of OBs. Finally, cells were cocultured inside a chamber and transplanted subcutaneously into immunodeficient mice to assess their in vivo bone‐forming capacity (Figure 1).

### 2.3. Isolation of HipOPs

Male C57BL/6 mice (6–8 weeks old) were euthanized via CO_2_ inhalation. In a sterile environment, femurs and tibiae were harvested, with their ends trimmed, and soaked in a cold phosphate‐buffered saline (PBS). Bone marrow tissue was flushed from the bone cavity using a syringe filled with culture medium (CM) and then gently agitated to create a single‐cell suspension. The CM was prepared by supplementing minimum essential medium (Thermo Fisher Scientific, Waltham, MA, USA) with 10% fetal bovine serum and 1% penicillin–streptomycin solution (Sigma–Aldrich, St. Louis, MO, USA). After straining to remove connective tissue, bone marrow cells were seeded in 10‐cm dishes at a density of 5 × 10^7^ cells/dish and cultured at 37°C in a humidified incubator with 5% CO_2_. Nonadherent cells were washed away on day 3 using PBS, while adherent cells were maintained in periodically refreshed CM. Once the cells reached ~80% confluence, they were harvested via enzymatic digestion with 0.25% trypsin‐EDTA solution (Sigma–Aldrich). In the purification stage by negative selection, cells were incubated with magnetic bead‐conjugated antibodies targeting hematopoietic cells, as described previously [19]. The mixture was passed through a magnetic field using a MACS separator (Miltenyi Biotec, Bergisch Gladbach, Germany), which bound and removed hematopoietic‐lineage cells. The remaining unlabeled cells were collected as HipOPs for further experiments.

### 2.4. OB Differentiation of HipOPs on Collagen Gel

The collagen gel solution was prepared using Cellmatrix type I‐A (Fujifilm Wako Pure Chemical, Tokyo, Japan) according to the manufacturer’s instructions. The solution (250 μL/well) was evenly distributed at the bottom of a 24‐well plate and incubated at 37°C for 30 min to solidify. Subsequently, 1 mL of HipOP suspension in CM (2.5 × 10^5^ cells) was added to each well, allowing cells to settle on the gel for 24 h. Osteoinduction was initiated by replacing the medium with osteoinductive medium (OM) containing ascorbic acid (50 μg/mL), β‐glycerophosphate (10^−3^ M), and dexamethasone (10^−8^ M). For comparison, BMSCs were included in parallel experiments. On days 1, 2, 3, 6, and 9, cells were fixed with 4% paraformaldehyde (PFA) for 20 min and subjected to alkaline phosphatase (ALP) and von Kossa staining to assess extracellular matrix formation on the collagen. Additionally, quantitative reverse transcription polymerase chain reaction (RT‐qPCR) was performed on duplicate samples to detect signaling molecules secreted by OBs during differentiation. These molecules included Wnt family proteins (Wnt1, 3a, 5a, 5b, 7b, and 10b), bone morphogenetic protein 2 (BMP2), and vascular endothelial growth factor A (VEGFA).

### 2.5. Coculture HipOPs and OBs in 3D Culture Model

To evaluate cell interactions in the 3D culture system, a confluent monolayer of OBs (10^5^ cells/well) was established on a collagen gel (50 µL/well) using a 96‐well plate. The even distribution of the OB layer was confirmed by a light microscope. Concurrently, hemostatic gelatin sponges (Spongel; LTL Pharma Co., Ltd., Tokyo, Japan) were prepared as cylinders (5 mm diameter and 3 mm height) using a biopsy punch. These sponges were sterilized with UV light before cell seeding. HipOP suspensions (10^6^ cells/50 µL) were seeded onto the sponges and incubated at 37°C for 2 h. Afterward, the sponges were transferred onto the OB layers in a 96‐well plate to establish the coculture model. Sponges cultured without the OB layer served as internal controls. After 48 h in CM, the sponges were bisected transversely under sterile conditions to investigate HipOP responses to signal concentration gradients. RNA was separately collected from the upper and lower halves of the sponges in both the HipOP/none group (no OB layer) and the HipOP/OB group (coculture with OBs) for gene expression profiling via RNA sequencing. Additionally, total RNA was extracted from intact sponges in each group to analyze osteogenic gene expression using RT‐qPCR, targeting markers such as *ALP*, *collagen type 1* (*Col-I*), *bone sialoprotein* (*BSP*), and *osteocalcin* (*OCN*). On day 7, cell‐seeded sponges were collected to assess proliferation via MTT assay and visualize osteogenic differentiation through ALP staining. For staining, the sponges were fixed in 4% PFA for 1 h, dehydrated overnight at 4°C in PBS containing 20% sucrose, embedded in an optimal cutting temperature compound (Sakura Finetek, Tokyo, Japan), and frozen at −80°C. Transverse sections (30 µm thickness) were prepared for analysis.

### 2.6. ALP and Von Kossa Staining

To detect ALP activity, the ALP substrate solution was freshly prepared by combining Fast Red Violet LB salt (Sigma–Aldrich) with a mixture of N, N‐dimethylformamide (NACALAI TESQUE, INC., Kyoto, Japan), naphthol AS‐MX phosphate (Sigma–Aldrich), and 0.2 M tris‐HCl buffer (pH 8.3). The solution was filtered and applied to samples for 40 min at room temperature. For phosphate deposition visualization via von Kossa staining, samples were removed from the ALP solution and treated with a 2.5% silver nitrate aqueous solution for 30 min. After each staining step, samples were washed three times with distilled water to remove excess dye. The stained sections were analyzed and imaged using a light microscope (BZ‐X810; KEYENCE, Osaka, Japan).

### 2.7. RNA Isolation, Complementary DNA (cDNA) Synthesis, and RT‐qPCR Analysis

Total RNA was extracted from cells using the FastGene RNA Basic Kit (Nippon Genetics, Tokyo, Japan) following the manufacturer’s instructions. RNA concentration and purity were assessed using a NanoDrop Spectrophotometer (Thermo Fisher Scientific). The extracted RNA was reverse‐transcribed into cDNA using the ReverTra Ace qPCR RT Master Kit (TOYOBO, Osaka, Japan). The cDNA samples were diluted in RNase/DNase‐free water as needed and used to prepare the PCR reaction mixture. This mixture contained THUNDERBIRD SYBR qPCR Mix (TOYOBO) and sequence‐specific primers (Thermo Fisher Scientific) as shown in Table 1. A 96‐well PCR plate was loaded with 20 µL of the reaction mixture per well and processed in an ABI 7500 Fast System (Thermo Fisher Scientific). Gene amplification and quantification were performed using the SYBR Green detection method. Data were analyzed using the 2^−ΔΔCt^ method to calculate the relative change in target gene expression, normalized to glyceraldehyde 3‐phosphate dehydrogenase (GAPDH) as the reference gene. Results were expressed as percentages relative to the highest value observed in the compared groups.

**Table 1:** Primer sequences of genes analyzed by RT‐qPCR.

Genes	Forward sequence (5′–3′)	Reverse sequence (5′–3′)
*Wnt1*	CGAGAGTGCAAATGGCAATTCCG	GATGAACGCTGTTTCTCGGCAG
*Wnt3a*	AACTGCACCACCGTCAGCAACA	AGCGTGTCACTGCGAAAGCTAC
*Wnt5a*	GGAACGAATCCACGCTAAGGGT	AGCACGTCTTGAGGCTACAGGA
*Wnt5b*	GCTACCGCTTTGCCAAGGAGTT	CATTTGCAGGCGACATCAGCCA
*Wnt7b*	TTCTCGTCGCTTTGTGGATGCC	CACCGTGACACTTACATTCCAGC
*Wnt10b*	ACCACGACATGGACTTCGGAGA	CCGCTTCAGGTTTTCCGTTACC
*BMP2*	AACACCGTGCGCAGCTTCCATC	CGGAAGATCTGGAGTTCTGCAG
*VEGFA*	CTGCTGTAACGATGAAGCCCTG	GCTGTAGGAAGCTCATCTCTCC
*ALP*	GGCCATCTAGGACCGGAGA	TGTCCACGTTGTATGTCTTGG
*Col-I*	GCTCCTCTTAGGGGCCACT	ATTGGGGACCCTTAGGCCAT
*BSP*	AAAATGGAGACGGCGATAGTT	GAGTGTGGAAAGTGTGGAGTTCT
*OCN*	GAGGACCATCTTTCTGCTCACT	CGGAGTCTGTTCACTACCTTATTG
*GAPDH*	AATGGATTTGGACGCATTGGT	TTTGCACTGGTACGTGTTGAT

### 2.8. In Vivo Transplantation Experiment

For transplantation, besides using collagen gel to support the OB layer and gelatin foam to seed HipOPs, we employed a perforated poly‐lactidecoglycolide (PLGA) tubular cell culture chamber (5 mm diameter × 5 mm height; GC Co., Tokyo, Japan) that was utilized as the outer framework due to its sufficient mechanical properties. These chambers featured circular holes of different sizes for specific purposes: small holes (0.5 mm diameter) at the base supported collagen attachment, while larger holes (2 mm diameter) above facilitated nutrient transport (Figure 1). Before use, the PLGA chambers were sterilized with 80% ethanol and placed in a 6‐well plate. Collagen solution (50 µL) was loaded into the chamber’s bottom and allowed to solidify before inducing osteogenic differentiation of HipOPs. Once a confluent OB monolayer on the collagen gel was confirmed, HipOP‐embedded sponges were added to complete the 3D model for the HipOP/OB group (Figure 1). For the HipOP/none group, the OB layer on collagen gel was omitted. These cell‐scaffold constructs were maintained in CM for 48 h before subcutaneous transplantation into immunodeficient mice (Crlj:CD1‐Foxn1nu mice). Samples were retrieved 8 weeks posttransplantation for further analysis.

### 2.9. Micro‐Computed Tomography (Micro‐CT) Analysis

After the sample retrieval, the mineralization in the transplants was first examined using the R‐mCT2 system (Rigaku, Tokyo, Japan) with a tube current of 160 μA, a tube voltage of 90 kV, and a slice width of 5 μm. TRI/3D‐BON‐FCS software (Ratoc System Engineering, Tokyo, Japan) was used to reconstruct 3D microstructures and calculate total mineralized tissue volume. A uniform threshold was applied to all specimens to distinguish mineralized tissue from the background.

### 2.10. Histological and Immunohistochemical Analysis

Retrieved transplants were fixed in 4% PFA at 4°C for 48 h and then decalcified in Kalkitox (Fujifilm Wako Pure Chemical) for 7 days with gentle agitation. Samples were dehydrated in a graded ethanol series, embedded in paraffin, and sectioned vertically into 7‐μm‐thick slices. For later analysis, the sections were gently dewaxed in xylene, rehydrated, and rinsed in distilled water. Hematoxylin–eosin (HE) staining was performed to observe general histological features, while Masson’s trichrome staining (Modified Masson’s; ScyTek Laboratories, West Logan, UT, USA) visualized bone matrix formation. For immunohistochemical staining of CD31, aimed at evaluating new blood vessel formation, antigen retrieval was performed by treating sections with citrate buffer (pH 6.0) at 80°C for 20 min. Nonspecific staining was blocked by incubating sections in a 10% normal goat serum solution in PBS for 1 h at room temperature. Sections were then incubated with anti‐CD31 antibody (ab182981; Abcam, Cambridge, UK) diluted 1:500 in blocking buffer and stored overnight in a humidified chamber at 4°C. Following PBS washing, sections were treated with a biotinylated secondary antibody for 30 min. Endogenous peroxidase activity was quenched using 0.3% hydrogen peroxide in methanol for 20 min, and color development was achieved with Elite ABC reagent (VECTASTAIN Elite ABC kit; Vector Laboratories, Newark, CA, USA) and diaminobenzidine substrate (ImmPACT DAB; Vector Laboratories). Finally, sections were counterstained with hematoxylin for nuclear visualization, dehydrated, and sealed with Mount‐Quick (Daido Sangyo Co., Tokyo, Japan). Stained sections were examined using a BZ‐810 microscope (KEYENCE).

### 2.11. Statistical Analysis

All experiments were conducted independently three times, and the results were presented as the means ± standard deviations. Comparisons between two groups were analyzed using the Student’s *t*‐test. For comparisons among more than two groups, the one‐way ANOVA was performed, followed by the post hoc Tukey’s HSD test to determine which specific groups differ from each other. All the statistical analysis was conducted using IBM SPSS Statistics 22 software (International Business Machines Co., Armonk, NY, USA). Differences were considered statistically significant when the *p*‐value was less than 0.05.

## 3. Results

### 3.1. OB Differentiation of HipOPs on Collagen Gel

Over the 9‐day osteoinduction period on collagen gel, the BMSC group displayed weak ALP activity beginning only at day 6 (Figure 2A). In contrast, HipOPs exhibited robust osteogenic differentiation, with ALP activity detectable from day 1 and calcium deposition starting from day 3 (Figure 2A). Both ALP staining and mineralized areas in the HipOP group expanded progressively over time, reaching their peaks at days 6 and 9, respectively (Figure 2A).

Figure 2Osteoblast differentiation of HipOPs on collagen gel. (A) Unfractionated BMSCs and HipOPs were cultured on a collagen gel, and practiced alkaline phosphatase (ALP) and von Kossa staining. ALP‐positive staining (red) marks early‐stage osteoblast differentiation, while calcium deposits in the bone matrix (black) indicate later‐stage differentiation. Scale bar: 200’ µm. (B, C) Relative gene expression levels of HipOPs during 9 days of osteogenic differentiation were normalized to the Ct value of the housekeeping gene *GAPDH* and presented as percentages relative to the highest value among the groups. One‐way ANOVA and Tukey’s HSD test, *n* = 3. Statistical significance is indicated as  ^∗^ *p* < 0.05,  ^∗∗^ *p* < 0.01,  ^∗∗∗^ *p* < 0.001.

**Figure:**
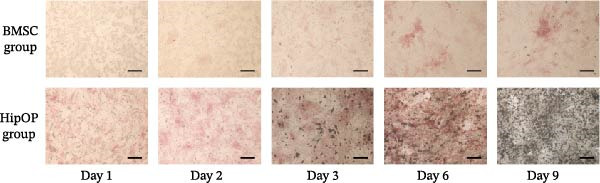
(A)

**Figure:**
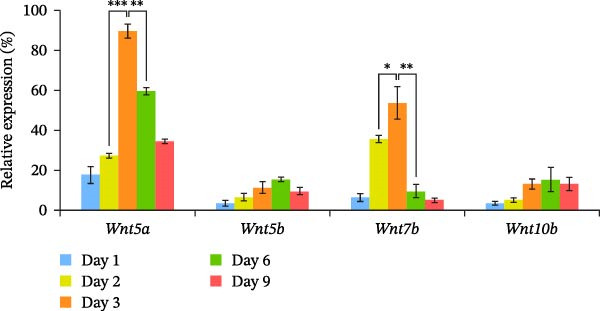
(B)

**Figure:**
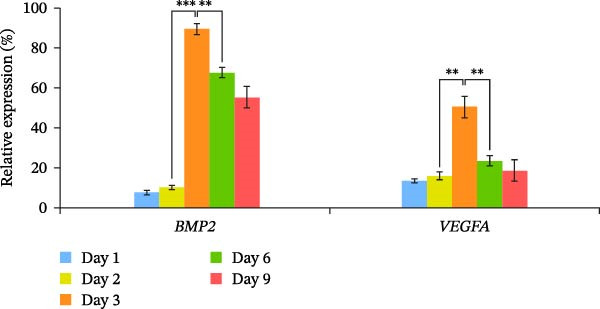
(C)

To monitor OB differentiation during the osteogenic induction process, gene expression profiles of the HipOP group were analyzed by RT‐qPCR on days 1, 2, 3, 6, and 9. Among the six key Wnt family genes studied, Wnt1 and Wnt3a were not expressed in the OB layer during the initial 9 days of differentiation. However, other Wnt family members, including Wnt5a, Wnt5b, Wnt7b, and Wnt10b, showed significant changes in gene expression as HipOPs differentiated into OBs. Specifically, data revealed significant upregulation of Wnt5a (*p* = 0.00001) and Wnt7b (*p* = 0.034) from day 2 to a peak on day 3, followed by a gradual downregulation (Figure 2B). With Wnt5b and Wnt10b, downregulated gene expression was only observed from day 6 of osteogenic differentiation. Similarly, growth factors BMP2 and VEGFA exhibited a comparable expression pattern, with significant upregulation from day 2 to day 3, peaking at day 3, and then gradually decreasing (Figure 2C). These findings suggested that mature OBs, which demonstrated positive von Kossa staining after 3 days of osteoinduction (Figure 2A). Given that the majority of the selected genes, including Wnt5a, Wnt7b, BMP2, and VEGFA, showed their most significant upregulation between days 2 and 3 before being downregulated (Figure 2B,C), the OB layer at day 2 in the HipOP group was used for subsequent experiments in the 3D culture model.

### 3.2. Effects of the OB Layer on HipOPs in the 3D Culture Model

**Figure 4:**
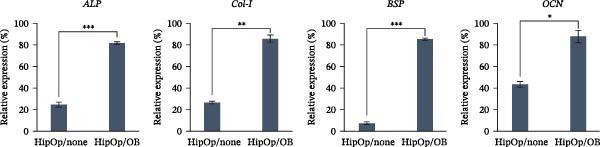
RT‐qPCR analysis of genes involved in bone formation. RNA was extracted from intact cell‐seeded sponges after 48 h of culture in both groups. Relative gene expression levels of bone formation‐related genes were normalized to the Ct value of the housekeeping gene *GAPDH* and presented as percentages relative to the highest value among the groups. Student’s *t*‐test, *n* = 3. Statistical significance is indicated as  ^∗^ *p* < 0.05,  ^∗∗^ *p* < 0.01,  ^∗∗∗^ *p* < 0.001.

RNA‐sequencing analysis was conducted to examine gene expression in HipOPs from the upper and lower halves of sponges. The data were represented as a *k*‐means clustering heatmap, a principal component analysis (PCA) plot, and scatter plots to visualize expression levels and similarities across groups. After 48 h of coculture with the OB layer, the HipOP population exhibited distinct gene expression profiles (Figure 3A–C). In cluster 1 of the *k*‐means clustering analysis (Figure 3A), Gene Ontology (GO) terms related to bone formation (Comp, Gpc3, Mepe, Smpd3, Actn3, and Cyp26b1), response to stimuli (Smad7, Ace, Col1a1, Igfbp2, Postn, and Ppl), cell communication (Adra1b, Gja3, Gjb3, Wnt4, and Wnt6), and regulation of vascular development (Flt1, Klf5, Hey2, Wt1, Myo18b, and Serpine1) were significantly enriched in the lower half of the sponge contacting the OB layer (Figure 3D). In contrast, the upper half of the HipOP/OB group displayed intermediate expression levels for these biological processes, while both halves of the HipOP/none group showed low expression levels (Figure 3A,D). Furthermore, gene expression profiles were consistent between the upper and lower halves in the HipOP/none group, indicating a lack of spatial differentiation (Figure 3A–C). RT‐qPCR analysis further demonstrated significant upregulation of osteogenic differentiation markers (*ALP*, *Col-I*, *BSP*, and *OCN*) in HipOPs after 48 h of coculture with the OB layer (Figure 4).

Figure 3RNA‐sequencing analysis of gene expression profiling. RNA was collected from HipOPs in the upper (U) and lower (L) halves of cell‐seeded sponges after 48 h of culture alone (HipOP/none group) or coculture with the OB layer (HipOP/OB group). (A) *k*‐means clustering heatmap of 2000 differentially expressed genes, where red indicates high expression and green indicates low expression. (B, C) Principal component analysis (PCA) and scatter plot showing the distribution of gene expression. PC1 accounts for the greatest variation in the data, while PC2 represents the second largest variation. The *R*‐value is the correlation coefficient. (D) Gene Ontology (GO) analysis of genes in cluster 1 from the *k*‐means clustering highlights the most enriched biological pathways, sorted by the false discovery rate (FDR). FDR values are derived from enrichment *p*‐values. Fold enrichment represents the overrepresentation of pathway‐related genes relative to the background. In the network plot, each pathway is depicted as a node, with size indicating gene count and color intensity representing statistical significance. Nodes sharing 30% or more genes are connected, with line thickness proportional to the number of overlapping genes.

**Figure:**
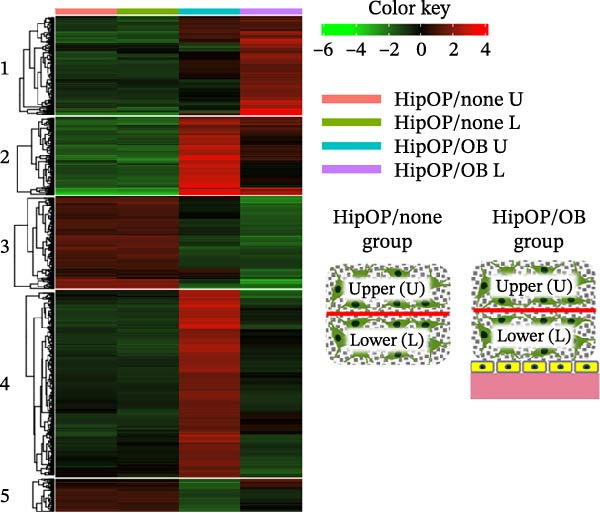
(A)

**Figure:**
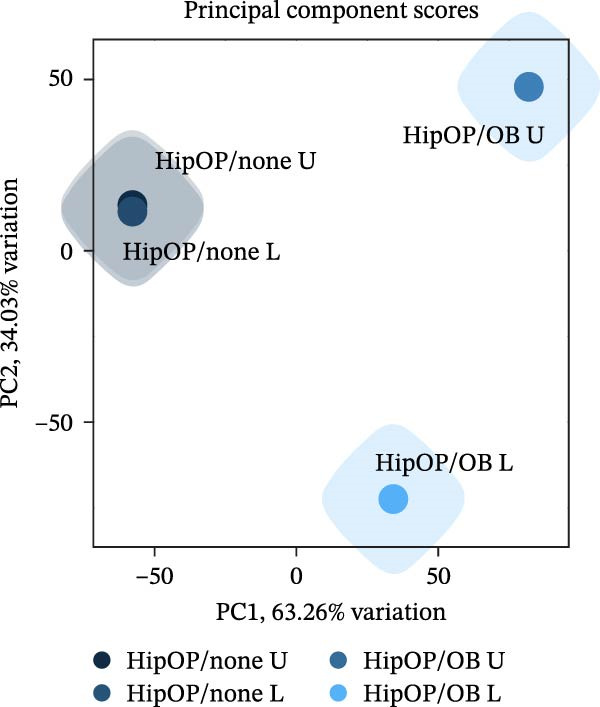
(B)

**Figure:**
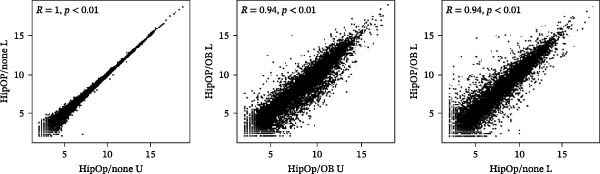
(C)

**Figure:**
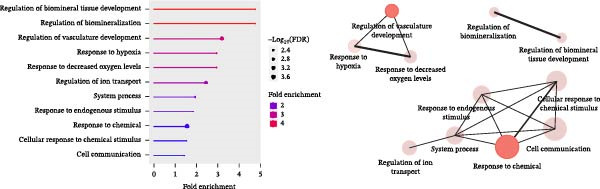
(D)

By day 7, ALP staining confirmed robust osteogenic differentiation in HipOP‐seeded sponges, with stronger staining intensity observed in the lower part of the sponge closer to the OB layer (Figure 5A, Supporting Information 1: Figure S1). Moreover, the lower half of the sponge in the HipOP/OB group showed a significantly higher number of cells compared to the upper half on day 7 (Figure 5B). These findings suggest that the OB layer provided signals that promoted osteogenic differentiation and proliferation.

Figure 5Cell differentiation and proliferation in gelatin sponges on day 7 of culture. (A) ALP staining on transverse sections of cell‐seeded sponges. Images were captured from the central transverse sections of the sponge, representing differentiation activity. Scale bar: 200 µm. (B) The comparison of cell number between the upper and lower halves in each group was performed using the Student’s *t*‐test (*n* = 3). Statistical significance is indicated as  ^∗∗∗^ *p* < 0.001.

**Figure:**
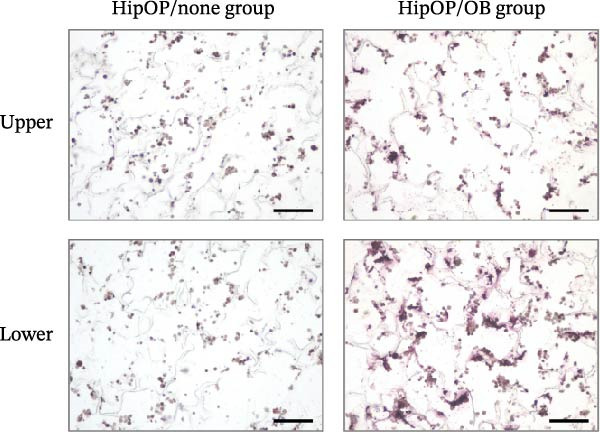
(A)

**Figure:**
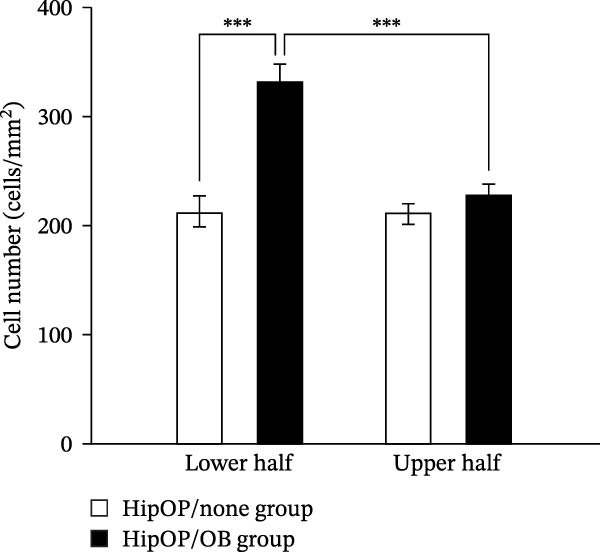
(B)

### 3.3. In Vivo Bone Formation Capacity of the 3D Culture Model

After the sample retrieval, the mineralization in the whole transplants was examined with Micro‐CT analysis. Micro‐CT analysis demonstrated the 3D reconstruction of mineralized tissue in both grayscale and pseudocolor, representing different bone density values. Data from the HipOP/OB group showed the formation of continuous bone‐like tissue. At the bottom, where the HipOP‐seeded sponge contacted the OB layer, a cortical bone‐like layer appeared with the highest density value (Figure 6A). The mineralization density gradually decreased from the bottom to the top. In contrast, the HipOP/none group exhibited two distinct ossification clusters in the lower and upper areas with irregular shapes and no density variation (Figure 6A). Additionally, the average bone volume in the HipOP/none group was significantly smaller than that in the HipOP/OB group (Figure 6B).

Figure 6Micro‐CT analysis after 8 weeks of transplantation. (A) 3D reconstruction images of mineralized tissue in grayscale and color gradients to represent different bone density values. (B) Comparison of generated bone volume between the HipOP/none group and the HipOP/OB group. Student’s *t*‐test, *n* = 3. Statistical significance is indicated as  ^∗∗^ *p* < 0.01.

**Figure:**
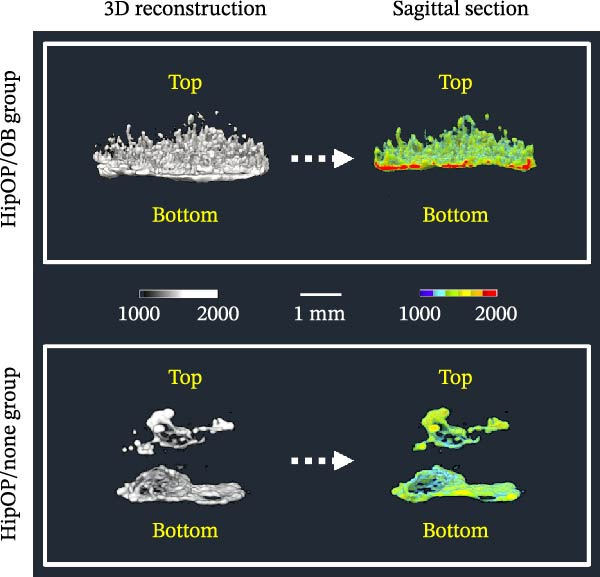
(A)

**Figure:**
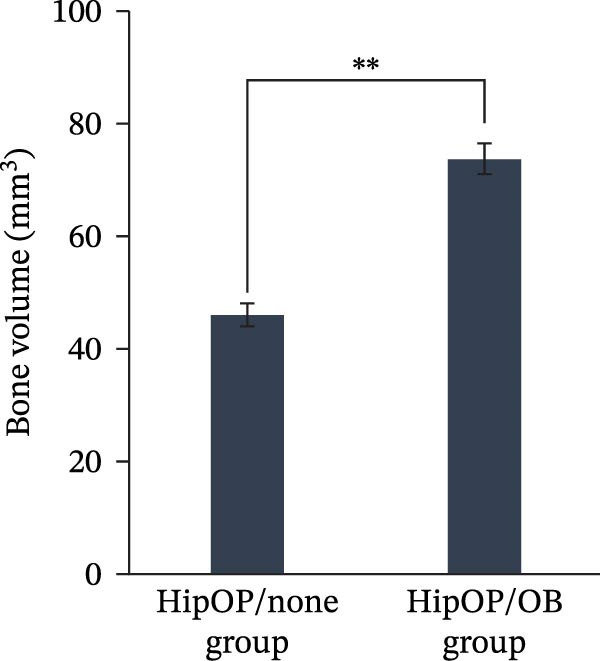
(B)

The bone density gradient in the HipOP/OB group was further assessed using Masson’s trichrome staining, which selectively stains collagen fibers in the bone matrix in different colors based on mineralization states. Newly formed bone, indicated in blue, was prominently present in the lower regions, while red appeared as a thin layer at the bottom end, suggesting mature bone tissue in this area (Figure 7A). However, samples in the HipOP/none group displayed positive staining only for the immature bone matrix. Bone matrix formation was observed beneath the bottom and top surfaces, becoming undetectable in the middle region (Figure 7A, Supporting Information 2: Figure S2). HE staining results confirmed the histology of the bone tissue in the HipOP/OB samples, showing the indications of OB and osteocyte phenotypes (Figure 7C). Ongoing Haversian canal formation was also noted within the cortical bone layer (Figure 7C). Furthermore, bone marrow tissue, including adipocytes, hematopoietic cells, blood vessels, and sinusoids, was identified in the bone cavity (Figure 7C). Immunohistochemical staining for CD31 revealed that the HipOP/OB group exhibited a highly developed vascular network throughout the lower half of the sample. Conversely, the HipOP/none group displayed minimal blood vessel formation (Figure 7B).

Figure 7Histological and immunohistochemical analysis of the transplants. (A) Masson’s trichrome staining on vertical sections visualizing bone matrix formation. Blue staining indicates collagen fibers and newly formed bone, while red staining marks mature bone. (B) Immunohistochemical staining of CD31, a marker for vascular endothelial cells, highlighting vascularization. (C) Hematoxylin and eosin (HE) staining of the HipOP/OB group showing spongy bone (sp), cortical bone (co), and ongoing Haversian canal (Hc) formation. Osteoblasts (red arrowheads) and osteocytes (black arrowheads) are visible in the bone matrix. Bone marrow tissue includes adipose tissue (red asterisks) and hematopoietic cells (he), which are nucleated and more basophilic than mature blood cells found in the sinusoid (sin). Scale bar for subparts (A) and (B): 200 µm.

**Figure:**
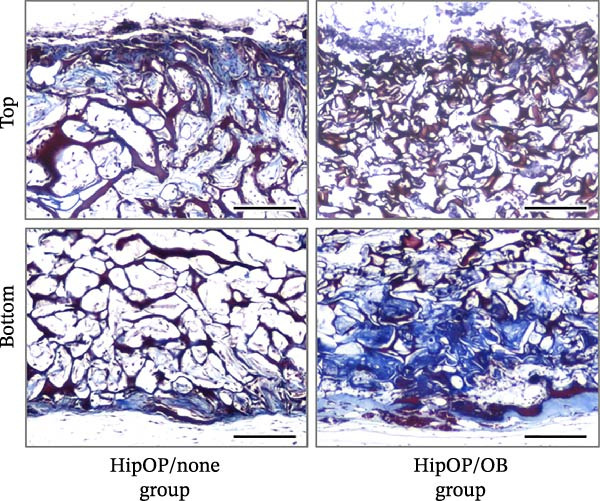
(A)

**Figure:**
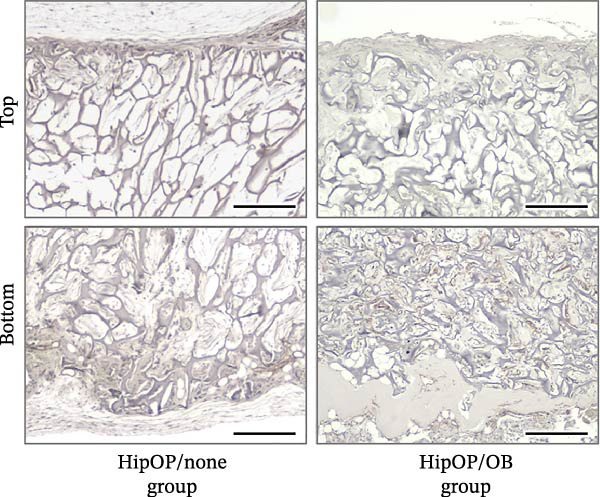
(B)

**Figure:**
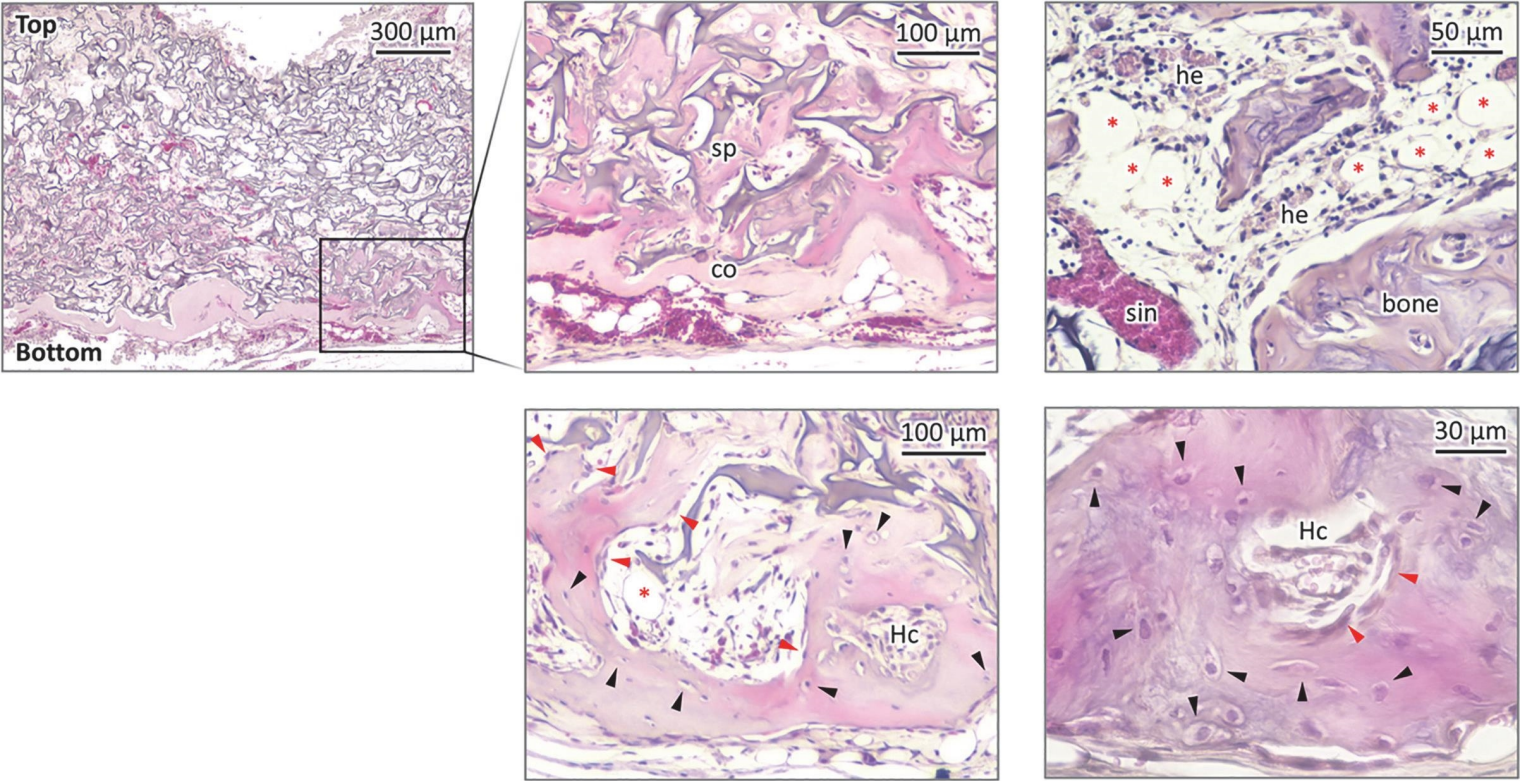
(C)

## 4. Discussion

One of the most important factors in regenerative medicine is a scaffold that mimics the extracellular matrix of the target tissue. In bone tissue engineering, collagen type 1, the primary component of the organic bone matrix, accounting for ~90%, has been a preferred scaffold material. Culturing BMSCs on a collagen gel promotes cell attachment, proliferation, and notably enhances osteogenic differentiation capacity [25–27]. In this study, when HipOPs were seeded on collagen gel and cultured in OM, the maturation of the OB layer occurred more rapidly compared to BMSCs in OM alone, as shown in a previous study [28]. Moreover, the collagen gel easily detached from the culture dish while remaining intact along the confluent OB layer. It also possessed sufficient tensile strength to support the HipOP‐sponge construct during delivery to the transplantation site. Similarly, gelatin, a denatured form of collagen with comparable biological properties and easier processing, could serve as a cost‐effective alternative for large‐scale bone regeneration applications. Researchers have utilized commercially available hemostatic gelatin sponges, known for their excellent pore interconnectivity, to facilitate nutrient exchange and 3D cell growth [29–31]. Finally, PLGA, a polymer with a long history of safe use in bone tissue engineering, is valued for its biocompatibility, biodegradability, and osteoconductivity. In this study, a new PLGA cell culture chamber was introduced, offering both physical support to prevent collagen gel contraction and mechanical strength to stabilize the entire model. By integrating these three materials, our model effectively leveraged OB‐derived signaling gradients to control the morphology of bone formation.

In addition, cells are another critical factor influencing the outcome of tissue engineering. Compared with untreated BMSCs, HipOPs demonstrated a higher osteogenic capacity [19]. The expression of ALP activity on day 1 indicated the presence of pre‐OBs in the HipOP population immediately after purification (Figure 2A), reflecting ongoing bone formation in the animal body prior to sacrifice [32]. As a heterogeneous cell population, HipOPs not only include multipotent stem cells but are also enriched with lineage‐committed cells, such as osteoprogenitors and pre‐OBs [19], which may accelerate the osteogenic differentiation process. In our experimental setup, osteogenic induction of HipOPs into mature OBs on collagen gel required only 3 days, as determined by OB maturational stages based on ALP activity and calcium deposits [33]. This rapid differentiation rate also stemmed from the removal of hematopoietic cells, which enhanced cell–cell communication, a critical factor for cellular differentiation [34]. Moreover, HipOPs were particularly advantageous in bone tissue regeneration, as they retained the capacity to support the bone marrow microenvironment and ensure functional blood cell formation [19].

Regarding growth factors in bone regeneration, previous studies demonstrated the osteogenic effects of OBs on BMSCs through paracrine signaling [9, 10, 35–37]. This study focused on well‐known members, including *Wnt1*, *3a*, *5a*, *5b*, *7b*, *10b*, and *BMP2*. Among these, neither *Wnt1* nor *Wnt3a* was detected during the 9 days of osteogenic induction (Figure 2B), consistent with previous observations [36]. *Wnt1* may only be expressed at later differentiation stages by late OBs and osteocytes [38]. Of the other Wnt ligands, Wnt10b is known to activate only the canonical pathway [39], while Wnt5a, Wnt5b, and Wnt7b are associated with both canonical and noncanonical pathways [40]. Which pathway is initiated depends on the concentration of Wnt ligands [41, 42], the differentiation state, and the activation threshold of the recipient cell [43]. Ultimately, both pathways serve essential roles in osteogenesis [40]. BMP signaling, another well‐established pathway, contributes to bone development independently or in conjunction with the Wnt pathway during osteogenic differentiation [44–46]. In this study, the influence of Wnt and BMP2 signaling on the HipOP population was suggested by the significant upregulation of osteogenic markers after 48 h of in vitro coculture (Figure 4) and the increased bone formation volume in the HipOP/OB group 2 months posttransplantation (Figure 6B).

Besides Wnts and BMP2, VEGF plays a critical role in bone regeneration. VEGFA is the most well‐known growth factor promoting endothelial cell proliferation, migration, and survival, essential for vascular development and angiogenesis [47–51]. In our 3D model, we observed that the OB layer served as a valuable source of VEGFA for endothelial cell migration and proliferation (Figure 2C). Although BMSCs and osteoprogenitors also produce VEGF [52], *VEGF* expression increases as cells differentiate toward OBs, as demonstrated in this study. Beyond its angiogenic role, VEGFA directly stimulates OB differentiation [51, 53] by upregulating the transcription factor RUNX2 [54]. The presence of OB‐derived VEGFA significantly influenced the angiogenesis observed between the HipOP/none and HipOP/OB groups following in vivo transplantation (Figure 7B). Adequate vascularization not only supports effective bone formation but also ensures the survival and functionality of regenerated bone in the defect area.

Similarly, the responses of the HipOP population to Wnts, BMP2, and VEGFA were reflected in enriched pathways associated with bone formation and vascular development, as revealed by RNA‐sequencing analysis (Figure 3D). However, these effects varied among cells within the sponge due to the diffusion of signaling molecules from the OB layer. Thus, HipOPs closer to the signal source might exhibit stronger changes. In this study, dividing the cell‐laden sponge in half to collect RNA for gene expression analysis could not fully verify the gradual transition of cellular responses from the bottom to the top. Nevertheless, a polarization in gene expression between the two halves of the sponge was observed in the HipOP/OB group (Figure 3A–C), supporting the fundamental mechanism of bone tissue formation with varying density values. Previous studies have reported that VEGF and BMP2 act as chemoattractants, facilitating the chemotactic migration of BMSCs [48, 55]. Similarly, our data demonstrated that OB‐derived signaling gradients involving Wnts, BMP2, and VEGFA first activated the HipOP response to these stimuli (Figure 3D) and subsequently promoted directional cell migration. This resulted in varying cell distributions within the sponge after 7 days of coculture (Figure 5). In the lower part, where the signaling was stronger, higher cell density likely enhanced cell communication and differentiation. In contrast, both halves of the sponge in the HipOP/none group exhibited equal increases in cell numbers but no differentiation activity, as indicated by negative ALP staining (Figure 5).

Therefore, in addition to the concentration‐dependent responses, cell migration is a secondary effect of the OB layer on HipOPs. It is essential to seed OBs in a 2D culture and HipOPs in a 3D culture to allow the soluble signals from the OB layer to form a gradient that directs bone formation by HipOPs. Cortical bone formation occurred only where the HipOP‐laden sponge was in contact with the OB layer. Although this pattern was absent in samples lacking the OB signaling gradient, bone still formed at the top and bottom. This outcome was attributed to passive condensation caused by the compression force of surrounding tissues and improved nutrient exchange in these areas of the HipOP/none samples. If our models are applied to bone defect reconstruction, the regenerated bone would seamlessly integrate with the host bone, restoring normal anatomy and original function. Consequently, bone remodeling—a slow process that can take years [56, 57]—would become unnecessary, significantly shortening the healing period.

## 5. Conclusions

Our proposed 3D culture model effectively utilized OB‐derived signaling molecules to direct cell migration and osteogenic differentiation of HipOPs, achieving bone regeneration with controlled morphology, including cortical bone, spongy bone, and bone marrow tissue.

## Author Contributions

Thi Hue Mai, Shousaku Itoh, and Takumi Kagioka developed the theory. Thi Hue Mai, Shousaku Itoh, Takumi Kagioka, Makato Abe, and Mikako Hayashi conceived the experiments. Thi Hue Mai, Shousaku Itoh, and Takumi Kagioka performed the experiments and analyzed the data. Thi Hue Mai, Shousaku Itoh, and Mikako Hayashi worked on the manuscript.

## Funding

The study was supported by the Japan Society for the Promotion of Science (Grants 24K02620 and 25K20282) and the Foundation Nakao Grants (Grant J230803006).

## Disclosure

The thesis related to this paper is opened on The University of Osaka Institutional Knowledge Archive [58]. All authors discussed the results and reviewed the final manuscript.

## Conflicts of Interest

The authors declare no conflicts of interest.

## Supporting Information

Additional supporting information can be found online in the Supporting Information section.

## Supporting information


**Supporting Information 1** Figure S1: The comparison of ALP‐positive cell number between the upper and lower halves in each group was performed using the Student’s *t*‐test (*n* = 3). Statistical significance is indicated as  ^∗∗∗^ *p* < 0.001.

**Supporting Information 2** Figure S2: HE staining of the full‐length sagittal section (vertically through the whole 3D coculture system) in vivo transplantation experiment of HipOP/none group. Scale bar for panels: 300 µm.

**Supporting Information 3** Figure S3: Immunohistochemical staining of control (A) and CD31 antibody (B). Scale bar for panels: 100 µm.

## Data Availability

The data that support the findings of this study are available from the corresponding author upon reasonable request.

## References

[1] Toosi S. , Javid-Naderi M. J. , Tamayol A. , Ebrahimzadeh M. H. , Yaghoubian S. , and Mousavi Shaegh S. A. , Additively Manufactured Porous Scaffolds by Design for Treatment of Bone Defects, Frontiers in Bioengineering and Biotechnology. (2024) 11, 10.3389/fbioe.2023.1252636. PMC10834686 38312510

[2] Tampieri A. , Porosity-Graded Hydroxyapatite Ceramics to Replace Natural Bone, Biomaterials. (2001) 22, no. 11, 1365–1370, 10.1016/S0142-9612(00)00290-8, 2-s2.0-0035371984. 11336309

[3] Hsu Y. H. , Turner I. G. , and Miles A. W. , Fabrication of Porous Bioceramics With Porosity Gradients Similar to the Bimodal Structure of Cortical and Cancellous Bone, Journal of Materials Science: Materials in Medicine. (2007) 18, no. 12, 2251–2256, 10.1007/s10856-007-3126-2, 2-s2.0-36448970143. 17562138

[4] Di Luca A. , Longoni A. , Criscenti G. , Mota C. , Van Blitterswijk C. , and Moroni L. , Toward Mimicking the Bone Structure: Design of Novel Hierarchical Scaffolds With a Tailored Radial Porosity Gradient, Biofabrication. (2016) 8, no. 4, 10.1088/1758-5090/8/4/045007, 2-s2.0-85006043008, 045007. 27725338

[5] Duan J. , Shao H. , and Liu H. , et al.3D Gel-Printing of Hierarchically Porous BCP Scaffolds for Bone Tissue Engineering, Journal of the European Ceramic Society. (2023) 43, no. 6, 2646–2653, 10.1016/j.jeurceramsoc.2023.01.010.

[6] Sun H. , Huang Y. , Zhang L. , Li B. , and Wang X. , Co-Culture of Bone Marrow Stromal Cells and Chondrocytes In Vivo for the Repair of the Goat Condylar Cartilage Defects, Experimental and Therapeutic Medicine. (2018) 16, 2969–2977, 10.3892/etm.2018.6551, 2-s2.0-85052704971. 30214515 PMC6125981

[7] Xu M. , Wani M. , and Dai Y. S. , et al.Differentiation of Bone Marrow Stromal Cells Into the Cardiac Phenotype Requires Intercellular Communication With Myocytes, Circulation. (2004) 110, no. 17, 2658–2665, 10.1161/01.CIR.0000145609.20435.36, 2-s2.0-7544237300. 15492307

[8] Kurenkova A. D. , Medvedeva E. V. , Newton P. T. , and Chagin A. S. , Niches for Skeletal Stem Cells of Mesenchymal Origin, Frontiers in Cell and Developmental Biology. (2020) 8, 10.3389/fcell.2020.00592. PMC7366157 32754592

[9] Tsai M. T. , Lin D. J. , Huang S. , Lin H. T. , and Chang W. H. , Osteogenic Differentiation is Synergistically Influenced by Osteoinductive Treatment and Direct Cell–Cell Contact Between Murine Osteoblasts and Mesenchymal Stem Cells, International Orthopaedics. (2012) 36, no. 1, 199–205, 10.1007/s00264-011-1259-x, 2-s2.0-84858795202. 21567150 PMC3251672

[10] Csaki C. , Matis U. , Mobasheri A. , and Shakibaei M. , Co-Culture of Canine Mesenchymal Stem Cells With Primary Bone-Derived Osteoblasts Promotes Osteogenic Differentiation, Histochemistry and Cell Biology. (2009) 131, no. 2, 251–266, 10.1007/s00418-008-0524-6, 2-s2.0-58149343768. 18941769

[11] Gaur D. , Yogalakshmi Y. , and Kulanthaivel S. , et al.Osteoblast-Derived Giant Plasma Membrane Vesicles Induce Osteogenic Differentiation of Human Mesenchymal Stem Cells, Advanced Biosystems. (2018) 2, no. 9, 10.1002/adbi.201800093, 2-s2.0-85065053795, 1800093.

[12] Cui Y. , Luan J. , Li H. , Zhou X. , and Han J. , Exosomes Derived From Mineralizing Osteoblasts Promote ST2 Cell Osteogenic Differentiation by Alteration of microRNA Expression, FEBS Letters. (2016) 590, no. 1, 185–192, 10.1002/1873-3468.12024, 2-s2.0-84955577585. 26763102

[13] Park J. S. , Yang H. N. , Woo D. G. , Kim H. , Na K. , and Park K. H. , Multi-Lineage Differentiation of hMSCs Encapsulated in Thermo-Reversible Hydrogel Using a Co-Culture System With Differentiated Cells, Biomaterials. (2010) 31, no. 28, 7275–7287, 10.1016/j.biomaterials.2010.06.006, 2-s2.0-77955281001. 20619450

[14] De Pace R. , Iaquinta M. R. , and Benkhalqui A. , et al.Revolutionizing Bone Healing: The Role of 3D Models, Cell Regeneration. (2025) 14, no. 1, 10.1186/s13619-025-00225-1. PMC11926310 40113735

[15] Brachtl G. , Poupardin R. , and Hochmann S. , et al.Batch Effects During Human Bone Marrow Stromal Cell Propagation Prevail Donor Variation and Culture Duration: Impact on Genotype, Phenotype and Function, Cells. (2022) 11, no. 6, 10.3390/cells11060946. PMC8946746 35326396

[16] Siddappa R. , Licht R. , van Blitterswijk C. , and de Boer J. , Donor Variation and Loss of Multipotency During In Vitro Expansion of Human Mesenchymal Stem Cells for Bone Tissue Engineering, Journal of Orthopaedic Research. (2007) 25, no. 8, 1029–1041, 10.1002/jor.20402, 2-s2.0-34447283203. 17469183

[17] Agata H. , Asahina I. , and Watanabe N. , et al.Characteristic Change and Loss of In Vivo Osteogenic Abilities of Human Bone Marrow Stromal Cells During Passage, Tissue Engineering Part A. (2010) 16, no. 2, 663–673, 10.1089/ten.tea.2009.0500, 2-s2.0-77049127376. 19754223

[18] Cai Y. , Liu T. , Fang F. , Xiong C. , and Shen S. , Comparisons of Mouse Mesenchymal Stem Cells in Primary Adherent Culture of Compact Bone Fragments and Whole Bone Marrow, Stem Cells International. (2015) 2015, 708906. 25821472 10.1155/2015/708906 PMC4363588

[19] Itoh S. and Aubin J. E. , A Novel Purification Method for Multipotential Skeletal Stem Cells, Journal of Cellular Biochemistry. (2009) 108, no. 2, 368–377, 10.1002/jcb.22262, 2-s2.0-70349235392. 19591175

[20] Colter D. C. , Class R. , DiGirolamo C. M. , and Prockop D. J. , Rapid Expansion of Recycling Stem Cells in Cultures of Plastic-Adherent Cells From Human Bone Marrow, Proceedings of the National Academy of Sciences. (2000) 97, no. 7, 3213–3218, 10.1073/pnas.97.7.3213, 2-s2.0-0034724369. PMC16218 10725391

[21] Kisiel A. , Mcduffee L. , Masaoud E. , Bailey T. , Esparza B. , and Nino Fong R. , Isolation, Characterization, and In Vitro Proliferation of Canine Mesenchymal Stem Cells Derived From Bone Marrow, Adipose Tissue, Muscle, and Periosteum, American Journal of Veterinary Research. (2012) 73, no. 8, 1305–1317, 10.2460/ajvr.73.8.1305, 2-s2.0-84865614168. 22849692

[22] Martin D. R. , Cox N. R. , Hathcock T. L. , Niemeyer G. P. , and Baker H. J. , Isolation and Characterization of Multipotential Mesenchymal Stem Cells From Feline Bone Marrow, Experimental Hematology. (2002) 30, no. 8, 879–886, 10.1016/S0301-472X(02)00864-0, 2-s2.0-0036327083. 12160839

[23] Song K. , Huang M. , Shi Q. , Du T. , and Cao Y. , Cultivation and Identification of Rat Bone Marrow-Derived Mesenchymal Stem Cells, Molecular Medicine Reports. (2014) 10, no. 2, 755–760, 10.3892/mmr.2014.2264, 2-s2.0-84903274614. 24859847

[24] Hu Y. , Lou B. , and Wu X. , et al.Comparative Study on In Vitro Culture of Mouse Bone Marrow Mesenchymal Stem Cells, Stem Cells International. (2018) 2018, 10.1155/2018/6704583, 2-s2.0-85056126504, 6704583. 29760732 PMC5924976

[25] Fernandes L. F. , Costa M. A. , Fernandes M. H. , and Tomás H. , Osteoblastic Behavior of Human Bone Marrow Cells Cultured Over Adsorbed Collagen Layer, Over Surface of Collagen Gels, and inside Collagen Gels, Connective Tissue Research. (2009) 50, no. 5, 336–346, 10.1080/03008200902855909. 19863393

[26] Mizuno M. , Fujisawa R. , and Kuboki Y. , Type I Collagen-Induced Osteoblastic Differentiation of Bone-Marrow Cells Mediated by Collagen-α2β1 Integrin Interaction, Journal of Cellular Physiology. (2000) 184, no. 2, 207–213, 10.1002/(ISSN)1097-4652. 10867645

[27] Vijayalekha A. , Anandasadagopan S. K. , and Pandurangan A. K. , An Overview of Collagen-Based Composite Scaffold for Bone Tissue Engineering, Applied Biochemistry and Biotechnology. (2023) 195, no. 7, 4617–4636, 10.1007/s12010-023-04318-y. 36652090

[28] Castano-Izquierdo H. , Álvarez-Barreto J. , van den Dolder J. , Jansen J. A. , Mikos A. G. , and Sikavitsas V. I. , Pre-Culture Period of Mesenchymal Stem Cells in Osteogenic Media Influences Their In Vivo Bone Forming Potential, Journal of Biomedical Materials Research Part A. (2007) 82A, no. 1, 129–138, 10.1002/jbm.a.31082, 2-s2.0-34250210761. 17269144

[29] Wang C. Y. , Kuo Z. K. , and Hsieh M. K. , et al.Cell Migration of Preosteoblast Cells on a Clinical Gelatin Sponge for 3D Bone Tissue Engineering, Biomedical Materials. (2019) 15, no. 1, 10.1088/1748-605X/ab4fb5, 015005. 31634880

[30] Kuo Z. K. , Lai P. L. , and Toh E. K. W. , et al.Osteogenic Differentiation of Preosteoblasts on a Hemostatic Gelatin Sponge, Scientific Reports. (2016) 6, no. 1, 10.1038/srep32884, 2-s2.0-84987660537, 32884. 27616161 PMC5018723

[31] Rohanizadeh R. , Swain M. , and Mason R. , Gelatin Sponges (Gelfoam) as a Scaffold for Osteoblasts, Journal of Materials Science: Materials in Medicine. (2008) 19, no. 3, 1173–1182, 10.1007/s10856-007-3154-y, 2-s2.0-40349102190. 17701305

[32] de Oliveira P. T. , Zalzal S. F. , Irie K. , and Nanci A. , Early Expression of Bone Matrix Proteins in Osteogenic Cell Cultures, Journal of Histochemistry & Cytochemistry. (2003) 51, no. 5, 633–641, 10.1177/002215540305100509. 12704211

[33] Bellosta P. , Masramon L. , Mansukhani A. , and Basilico C. , p21WAF1/CIP1 Acts as a Brake in Osteoblast Differentiation, Journal of Bone and Mineral Research. (2003) 18, no. 5, 818–826, 10.1359/jbmr.2003.18.5.818, 2-s2.0-0142027569. 12733720

[34] Schiller P. C. , D’Ippolito G. , Balkan W. , Roos B. A. , and Howard G. A. , Gap-Junctional Communication is Required for the Maturation Process of Osteoblastic Cells in Culture, Bone. (2001) 28, no. 4, 362–369, 10.1016/S8756-3282(00)00458-0, 2-s2.0-0035032495. 11336916

[35] Glueck M. , Gardner O. , and Czekanska E. , et al.Induction of Osteogenic Differentiation in Human Mesenchymal Stem Cells by Crosstalk Wth Osteoblasts, BioResearch Open Access. (2015) 4, no. 1, 121–130, 10.1089/biores.2015.0002, 2-s2.0-84960113928. 26309789 PMC4497645

[36] Wang Y. , Volloch V. , Pindrus M. A. , Blasioli D. J. , Chen J. , and Kaplan D. L. , Murine Osteoblasts Regulate Mesenchymal Stem Cells via WNT and Cadherin Pathways: Mechanism Depends on Cell–Cell Contact Mode, Journal of Tissue Engineering and Regenerative Medicine. (2007) 1, no. 1, 39–50, 10.1002/term.6, 2-s2.0-42949140534. 18038391

[37] Takeuchi R. , Katagiri W. , Endo S. , and Kobayashi T. , Exosomes From Conditioned Media of Bone Marrow-Derived Mesenchymal Stem Cells Promote Bone Regeneration by Enhancing Angiogenesis, PLoS ONE. (2019) 14, e0225472. 31751396 10.1371/journal.pone.0225472 PMC6872157

[38] Joeng K. S. , Lee Y. C. , and Lim J. , et al.Osteocyte-Specific WNT1 Regulates Osteoblast Function During Bone Homeostasis, Journal of Clinical Investigation. (2017) 127, no. 7, 2678–2688, 10.1172/JCI92617, 2-s2.0-85021778151. 28628032 PMC5490765

[39] Pederson L. , Ruan M. , Westendorf J. J. , Khosla S. , and Oursler M. J. , Regulation of Bone Formation by Osteoclasts Involves Wnt/BMP Signaling and the Chemokine Sphingosine-1-Phosphate, Proceedings of the National Academy of Sciences. (2008) 105, no. 52, 20764–20769, 10.1073/pnas.0805133106, 2-s2.0-58549115903. PMC2603259 19075223

[40] Lojk J. and Marc J. , Roles of Non-Canonical Wnt Signalling Pathways in Bone Biology, International Journal of Molecular Sciences. (2021) 22, no. 19, 10.3390/ijms221910840. PMC8509327 34639180

[41] van Amerongen R. , Fuerer C. , Mizutani M. , and Nusse R. , Wnt5A Can Both Activate and Repress Wnt/β-Catenin Signaling During Mouse Embryonic Development, Developmental Biology. (2012) 369, no. 1, 101–114, 10.1016/j.ydbio.2012.06.020, 2-s2.0-84864352822. 22771246 PMC3435145

[42] Grumolato L. , Liu G. , and Mong P. , et al.Canonical and Noncanonical Wnts use a Common Mechanism to Activate Completely Unrelated Coreceptors, Genes & Development. (2010) 24, no. 22, 2517–2530, 10.1101/gad.1957710, 2-s2.0-78349276165. 21078818 PMC2975928

[43] Quarto N. , Behr B. , and Longaker M. T. , Opposite Spectrum of Activity of Canonical Wnt Signaling in the Osteogenic Context of Undifferentiated and Differentiated Mesenchymal Cells: Implications for Tissue Engineering, Tissue Engineering Part A. (2010) 16, no. 10, 3185–3197, 10.1089/ten.tea.2010.0133, 2-s2.0-77957666829. 20590472 PMC2947420

[44] Huang W. , Yang S. , Shao J. , and Li Y. P. , Signaling and Transcriptional Regulation in Osteoblast Commitment and Differentiation, Frontiers in Bioscience. (2007) 12, no. 8–12, 3068–3092, 10.2741/2296, 2-s2.0-34347257781. 17485283 PMC3571113

[45] Itasaki N. and Hoppler S. , Crosstalk Between Wnt and Bone Morphogenic Protein Signaling: A Turbulent Relationship, Developmental Dynamics. (2010) 239, no. 1, 16–33, 10.1002/dvdy.22009, 2-s2.0-73949136201. 19544585

[46] Thomas S. and Jaganathan B. G. , Signaling Network Regulating Osteogenesis in Mesenchymal Stem Cells, Journal of Cell Communication and Signaling. (2022) 16, no. 1, 47–61, 10.1007/s12079-021-00635-1. 34236594 PMC8688675

[47] Ferrara N. , Gerber H. P. , and LeCouter J. , The Biology of VEGF and Its Receptors, Nature Medicine. (2003) 9, no. 6, 669–676, 10.1038/nm0603-669, 2-s2.0-0037699954. 12778165

[48] Dreyer C. H. , Kjaergaard K. , Ding M. , and Qin L. , Vascular Endothelial Growth Factor for In Vivo Bone Formation: A Systematic Review, Journal of Orthopaedic Translation. (2020) 24, 46–57, 10.1016/j.jot.2020.05.005. 32642428 PMC7334443

[49] Wernike E. , Montjovent M. O. , and Liu Y. , et al.VEGF Incorporated into Calcium Phosphate Ceramics Promotes Vascularisation and Bone Formation In Vivo, European Cells and Materials. (2010) 19, 30–40, 10.22203/eCM.v019a04. 20178096

[50] Grosso A. , Burger M. G. , Lunger A. , Schaefer D. J. , Banfi A. , and Di Maggio N. , It Takes Two to Tango: Coupling of Angiogenesis and Osteogenesis for Bone Regeneration, Frontiers in Bioengineering and Biotechnology. (2017) 5, 10.3389/fbioe.2017.00068, 2-s2.0-85037042869. PMC5675838 29164110

[51] Zelzer E. , McLean W. , and Ng Y. S. , et al.Skeletal Defects in VEGF120/120 Mice Reveal Multiple Roles for VEGF in Skeletogenesis, Development. (2002) 129, no. 8, 1893–1904, 10.1242/dev.129.8.1893. 11934855

[52] Phinney D. G. , Biochemical Heterogeneity of Mesenchymal Stem Cell Populations: Clues to Their Therapeutic Efficacy, Cell Cycle. (2007) 6, no. 23, 2884–2889, 10.4161/cc.6.23.5095, 2-s2.0-37549053263. 18000405

[53] Deckers M. M. L. , Karperien M. , Yamashita T. , and Papapoulos S. E. , Expression of Vascular Endothelial Growth Factors and Their Receptors During Osteoblast Differentiation, Endocrinology. (2000) 141, no. 5, 1667–1674, 10.1210/endo.141.5.7458, 2-s2.0-0034457236. 10803575

[54] Liu Y. , Berendsen A. D. , and Jia S. , et al.Intracellular VEGF Regulates the Balance Between Osteoblast and Adipocyte Differentiation, Journal of Clinical Investigation. (2012) 122, no. 9, 3101–3113, 10.1172/JCI61209, 2-s2.0-84866001590. 22886301 PMC3428080

[55] Zhang W. , Zhu C. , and Wu Y. , et al.VEGF and BMP-2 Promote Bone Regeneration by Facilitating Bone Marrow Stem Cell Homing and Differentiation, European Cells and Materials. (2014) 27, 1–12, 10.22203/eCM.v027a01, 2-s2.0-84892563631. 24425156

[56] ElHawary H. , Baradaran A. , and Abi-Rafeh J. , et al.Bone Healing and Inflammation: Principles of Fracture and Repair, Seminars in Plastic Surgery. (2021) 35, no. 3, 198–203, 10.1055/s-0041-1732334. 34526868 PMC8432998

[57] Siska P. A. , Gruen G. S. , and Pape H. C. , External Adjuncts to Enhance Fracture Healing: What is the Role of Ultrasound?, Injury. (2008) 39, no. 10, 1095–1105, 10.1016/j.injury.2008.01.015, 2-s2.0-50249095071. 18417130

[58] Mai T. H. , A Novel 3D Culture Model for Bone Regeneration With Controlled Morphology, The University of Osaka Institutional Knowledge Archive, https://ir.library.osaka-u.ac.jp/repo/ouka/all/101557/34737_Abstract.pdf.

