# Supplementary figures and images for: A Scaffold‐Based 3D Culture Model Including Selected Osteoprogenitors for Bone Regeneration With Controlled Morphology
Source: Stem Cells Int. 2026 Apr 10;2026:1860064. doi: 10.1155/sci/1860064 (PMC13066912; doi:10.1155/sci/1860064)

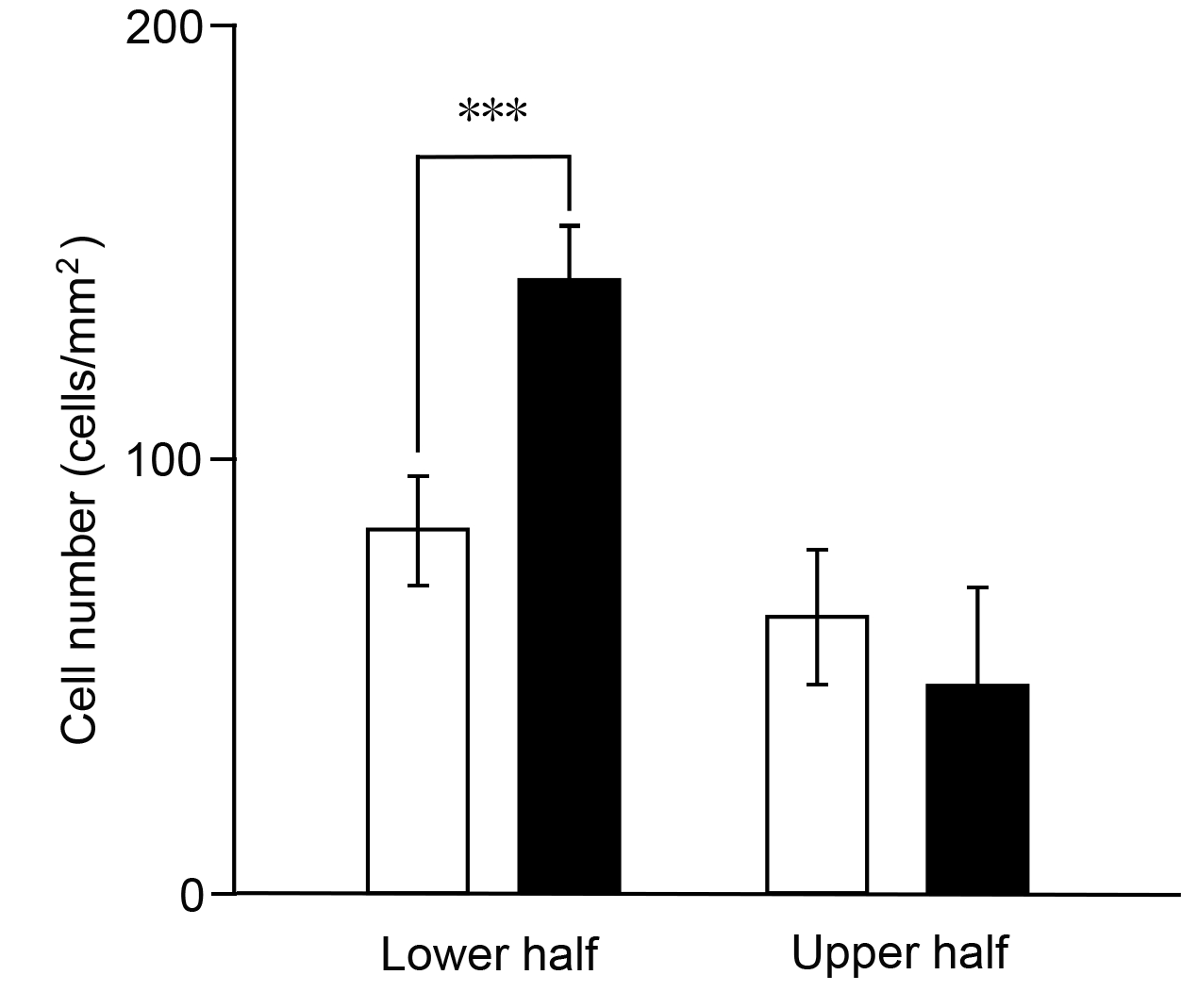

Supplement: Supplementary file 1 — Supporting Information 1 Figure S1: The comparison of ALP‐positive cell number between the upper and lower halves in each group was performed using the Student’s t‐test (n = 3). Statistical significance is indicated as ∗∗∗ p < 0.001. [file SCI-2026-1860064-s003.png]

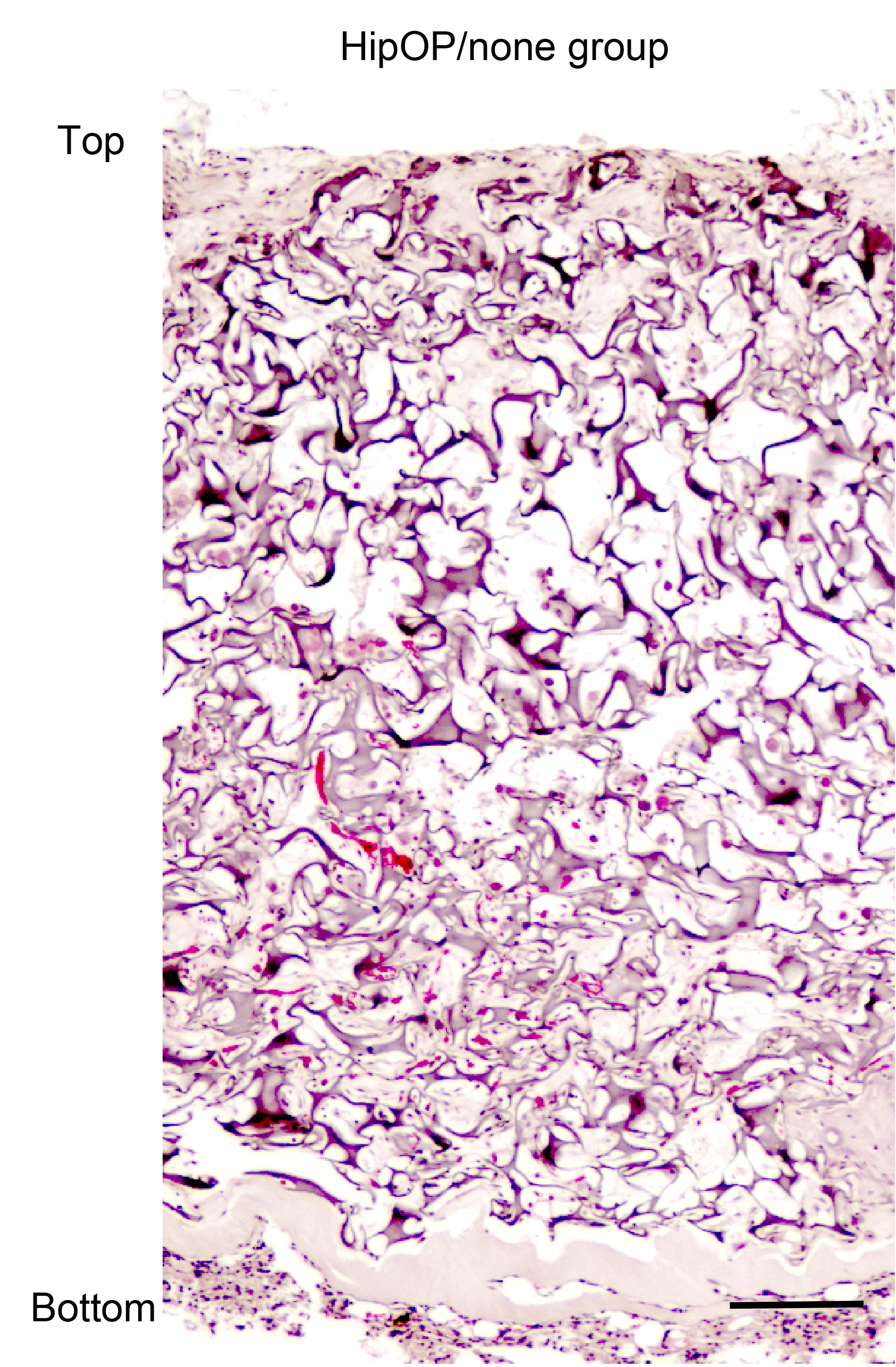

Supplement: Supplementary file 2 — Supporting Information 2 Figure S2: HE staining of the full‐length sagittal section (vertically through the whole 3D coculture system) in vivo transplantation experiment of HipOP/none group. Scale bar for panels: 300 µm. [file SCI-2026-1860064-s001.png]

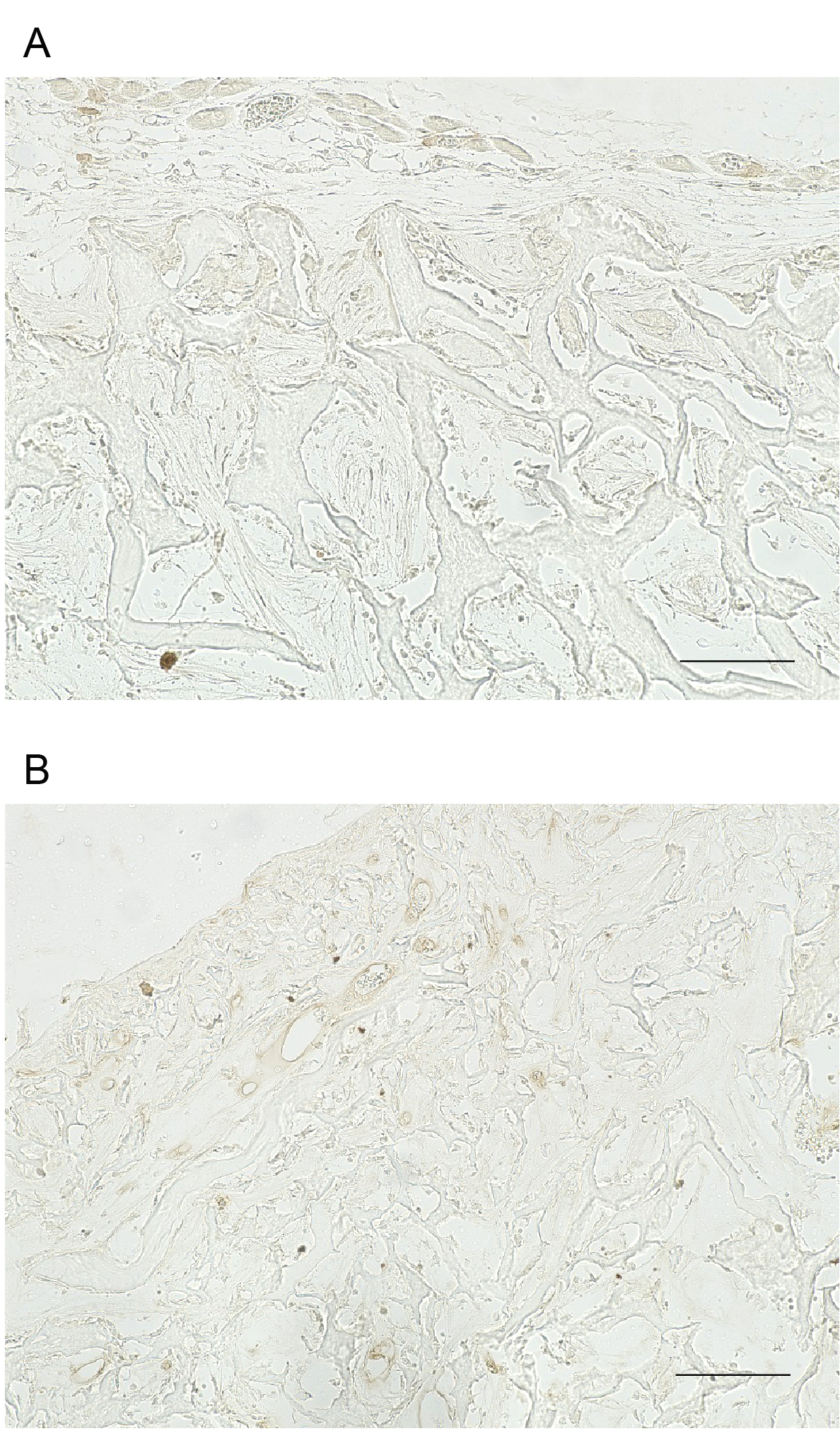

Supplement: Supplementary file 3 — Supporting Information 3 Figure S3: Immunohistochemical staining of control (A) and CD31 antibody (B). Scale bar for panels: 100 µm. [file SCI-2026-1860064-s002.png]
